# Mental Health and Psychotropic Stigma Among Student Pharmacists

**DOI:** 10.3389/fpubh.2022.818034

**Published:** 2022-03-28

**Authors:** Brandy Davis, Cassidi C. McDaniel, Chih-hsuan Wang, Kimberly B. Garza

**Affiliations:** ^1^Health Outcomes Research and Policy, Auburn University Harrison College of Pharmacy, Auburn, AL, United States; ^2^Educational Foundations, Leadership, and Technology, Auburn University, Auburn, AL, United States

**Keywords:** student, pharmacist, mental health, stigma, psychotropic

## Abstract

**Objective:**

To gain a better understanding of student pharmacists' stigma toward mental health and psychotropic medications.

**Methods:**

A cross-sectional study was conducted *via* paper and online surveys amongst all student pharmacists enrolled in a Doctor of Pharmacy program in the Southeastern United States (*n* = 501). The Perceived Devaluation and Discrimination (PDD) Scale was used to measure mental health stigma. The Beliefs about Medicines Questionnaire (BMQ) was modified to measure psychotropic stigma. MANOVAs were conducted to investigate relationships between student pharmacists' characteristics with mental health and psychotropic stigma. A paired *t*-test was used to determine if there was a difference between degree of mental health stigma and psychotropic stigma.

**Results:**

A total of 390 participants completed the survey (65%). The sample was mostly female (67%), white (79%), and non-Hispanic (96%). Ages were predominantly within the 19–24-year range (80%), and the majority of respondents reported previous interactions with patients who have mental health conditions (55%) or patients on psychotropic medications (65%). Student personal preferences for mental health treatment were primarily psychologic (42%) or both psychologic and psychotropic (40%). Degree of psychotropic stigma was significantly greater than that of mental health stigma. A statistically significant association was found between student personal preference for treatment and the psychotropic stigma. No difference was found in degree of either type of stigma across cohorts.

**Conclusions:**

Student pharmacists demonstrated both mental health and psychotropic stigmas. Future research should be performed to determine what effects these stigmas have on care of patients with mental health conditions.

## Introduction

As of 2019, one out of every five people are affected by a mental health condition (1, 2). Of these, over 70% do not receive treatment (3). Even when people do receive treatment, it can be delayed for up to a decade after the onset of symptoms (4, 5). When mental health conditions are left untreated, deleterious effects occur, such as decreased work productivity, school dropouts, and suicide (1, 2, 6, 7). Furthermore, improperly treated mental health conditions lead to increased hospitalizations, increasing the overall cost of care (2). In the United States, the effects of mental health conditions have resulted in a staggering $193.2 billion in lost earnings every year (2). Depression alone causes annual losses in productivity that cost the United States over $31 billion (5). Accordingly, the World Health Organization (WHO) has declared depression to be the leading cause of disability and poor health worldwide (8).

One way organizations such as Walgreens Company, the National Council for Behavioral Health, and the College of Psychiatric and Neurologic Pharmacists are combating this problem is to use pharmacists to bridge the mental healthcare gap (9, 10). Pharmacists are highly trained health providers that can help facilitate patient access to mental health providers while also providing medication counseling to this population. Pharmacists have been shown to improve mental health outcomes (11–15), and to be easily accessible within the United States (16, 17). However, pharmacists may lack confidence in their skills and knowledge of mental health conditions and treatments and have been reported to have stigma toward these patients (18, 19). Similarly, student pharmacists show stigma toward patients with mental health conditions like schizophrenia and severe depression that does not always lessen with years in pharmacy school (20).

While mental health stigma has been studied extensively (21, 22), psychotropic stigma has not, especially in student pharmacists. Stigma toward psychotropic medications can cause feelings of discomfort and judgement in patients taking psychotropic medications (23). It is possible that student pharmacists may also experience these feelings, and this may affect patient care in future practice. Hence, the objective of this study was to gain a better understanding of student pharmacists' experiences with stigma toward mental health and psychotropic medications. The following research questions were proposed to achieve the objective: (1) are there differences between degree of student pharmacists' stigma toward mental health conditions and stigma toward psychotropic medications; (2) are student pharmacists' stigma toward mental health conditions and psychotropic medications associated with personal preference of mental health treatment options (counseling, medication, both, neither); and (3) do student pharmacists' stigma toward mental health conditions and psychotropic medications differ between cohorts of student pharmacists?

## Methods

### Study Design

This cross-sectional study was conducted *via* both paper and online surveys amongst a sample of professional students enrolled in the Doctor of Pharmacy program, hereafter referred to as student pharmacists. The study occurred at a School of Pharmacy located in the Southeast. The protocol for study procedures was approved by the primary author's institutional review board.

### Participants and Recruitment

The Pharm.D. program at the primary author's institution consists of 4 years of coursework and training. Curricular content related to mental health conditions and their treatment include depression (introduced in the first year and reinforced in the second and third years), generalized anxiety disorder (introduced in the second year and reinforced in the third year), and schizophrenia (introduced in the second year and reinforced in the third year). The fourth year of training is experiential, and students are exposed to mental health-related topics depending upon which clinical rotation sites they are assigned. Stigma is introduced early in the curriculum, in the first semester of the first year of study. In fall semester of 2019, student pharmacists in all four cohorts were asked to voluntarily participate in this study to understand their perspectives of mental health conditions and psychotropic stigma. To be eligible for recruitment, student pharmacists were required to be enrolled in the Pharm.D. program at the School of Pharmacy and be over the age of 19. Through the Pharm.D. program, first-, second-, and third-year student pharmacists are primarily involved in the didactic portion of the curriculum, while fourth-year student pharmacists are involved in experiential learning at practice sites across the state. Based on these geographical differences, first-, second-, and third-year students completed a paper survey, while fourth-year students completed an online survey.

### Procedures

Student pharmacists in their first (*n* = 137, 35.1%), second (*n* = 142, 36.4%), and third (*n* = 98, 25.1%) year of the program were informed about the study in person and asked to complete a paper version of the anonymous survey at the end of a class period. An informed consent document was distributed to each student in the class. Students were provided with a verbal description of the study and consented in group settings. They were instructed to return the paper surveys, whether completed or not, in a sealed envelope to minimize coercion for participation. Based on different teaching modalities for each year, first- and third-year student pharmacists were recruited and participated in split classrooms, and second-year student pharmacists were recruited and participated in one large classroom. Lastly, fourth-year student pharmacists (*n* = 13, 3.3%) were recruited through email invitations to complete an online version of the anonymous Qualtrics survey, with information pertaining to informed consent electronically presented in the form of an information letter at the beginning of the survey. All survey items were identical to those included in the paper survey. In an effort to maximize participation from this less accessible group, fourth-year students who completed the survey were offered a chance to be entered in a drawing to win one of four $10 Amazon gift cards.

### Survey Development and Measures

The survey included items related to prior interaction with patients who have mental health conditions or who take psychotropic medications, personal mental health treatment, and personal psychotropic medication use. Two previously published scales were used to measure mental health and psychotropic stigma. First, the Perceived Devaluation Discrimination (PDD) Scale includes 12 items to indirectly measure mental health stigma (24). This measure has previously been validated (α = 0.86-0.88) and tested for reliability with a test-retest reliability of 0.93 (25). Second, the Beliefs About Medicines Questionnaire (BMQ)—General includes eight items that were modified and used to measure psychotropic stigma (26). The BMQ-General was developed to assess perspectives of general medication use; modifications to wording were used to assess perspectives of psychotropic medications specifically. Thus, for each item in the questionnaire, the word “psychotropic” was added preceding the word “medicine(s).” Additional survey items included areas of mental health and psychotropic medication stigmas related to job capabilities, diagnosis, and comfort communicating information with patients.

Items for the PDD Scale, modified BMQ-General, and our self-developed survey items were all measured on a five-point Likert-type scale from 1 indicating “Strongly Disagree” to 5 indicating “Strongly Agree.” Negatively worded items were reverse coded prior to analysis. Mental health stigma scores were obtained by adding the 12 item responses from the PDD Scale and dividing by 12 to obtain a mean score. Likewise, psychotropic stigma scores were obtained by adding the 8 item responses from the modified BMQ-General and dividing by 8 to obtain a mean score. Higher scores for mental health stigma and psychotropic stigma indicate lower degree of stigma, while lower scores indicate greater degree of stigma.

### Statistical Analysis

The psychometrics for the scales within the survey were assessed, which included validity and reliability assessments where appropriate. Both the PDD Scale and BMQ-general are validated tools (24, 26). Confirmatory Factor Analysis (CFA) was conducted to examine the construct validity of both instruments. A non-significant Goodness-of-fit Chi-square result is desired with the Chi-square/df is smaller than 5. The model fit indices, GFI, CFI, and TLI, are expected to be larger than 0.90, while the RMSEA is smaller than 0.08 (27). Reliability was evaluated using Cronbach's alpha for both the PDD Scale and the modified BMQ-General.

Student pharmacists' characteristics were analyzed using frequencies and included student's age, gender, race, ethnicity, year in pharmacy school, personal mental health and psychotropic medication taking, and experiences interacting with patients about mental health and/or psychotropic medications. The mental health stigma score, psychotropic stigma score, and miscellaneous stigma items were analyzed using means and standard deviations (SD). To compare means from mental health stigma and psychotropic stigma, paired t-test was used. To determine whether mental health stigma and psychotropic stigma was associated with personal preference of mental health treatment options (counseling, medication, both, neither), an additional MANOVA was conducted with mental health stigma and psychotropic stigma as the two dependent variables, but the independent variable was the student's treatment preference for mental health conditions categorized as counseling, medication, both, or neither. For both MANOVA tests, the assumptions of multivariate normal distribution in each group, homoscedasticity, and linear relationship among dependent variables were met, but the assumption of random sampling was violated because the sample was purposefully collected from this group of student pharmacists. To determine whether there were differences in mental health stigma and psychotropic stigma between cohorts of student pharmacists, a multivariate analysis of variance (MANOVA) was conducted with mental health stigma and psychotropic stigma as the two dependent variables, and the independent variable was the students' year in the Pharm.D. program. Statistical significance was set at 0.05, and analyses were performed using SPSS and AMOS, Version 25.

## Results

### Participant Characteristics

A total of 390 student pharmacists participated by responding to the anonymous survey, resulting in a 93% response rate for first year student pharmacists, 94% response rate for second year student pharmacists, 64% response rate for third year student pharmacists, 9% response rate for fourth year student pharmacists and a total response rate of 65% (Table 1). Most student pharmacists were aged 19-24 years old (80%), female (67%), white (79%), and not Hispanic (96%). Distribution of respondents across years in pharmacy school ranged from 137 first-year students, 142 second-year students, 98 third-year students, and 13 fourth-year students. The majority of student pharmacists had experiences interacting with patients who had mental health problems (55.1%) or were taking psychotropic medications (65.1%). As for students' personal history with mental health or psychotropics, only about 25% of student pharmacists were reported currently receiving or having previously received counseling from a certified counselor or psychologist. Additionally, only about 25% of student pharmacists reported currently taking psychotropics or having taken them in the past. Student personal preference for mental health treatment varied with the majority preferring counseling (41.8%) or both counseling and medication (39.7%). Few preferred only medication (14.4%) and even fewer preferred no treatment at all (3.3%).

**Table 1:** Participant characteristics (*N* = 390).

	***n* (%)**
Female	261 (66.9)
**Age**	
19-24	311 (80)
25-34	72 (18.5)
35-44	6 (1.5)
**Race**	
White	306 (78.5)
Black	37 (9.5)
Asian	30 (7.7)
Other	17 (4.3)
**Ethnicity**	
Not hispanic	376 (96.4)
**Year in Pharm.D. program**	
1	137 (35.1)
2	142 (36.4)
3	98 (25.1)
4	13 (3.4)
**Student experiences**	
Interacted with patients who have mental health problems	215 (55.1)
Interacted with patients who are taking psychotropic medications	254 (65.1)
**Personal treatment experience**	
Ever taken psychotropics	97 (24.9)
Ever received counseling	96 (24.6)
**Personal treatment preference**	
Prefers counseling	163 (41.8)
Prefers medication	56 (14.4)
Prefers both	155 (39.7)
Prefers neither	13 (3.3)

### Psychometrics

#### Validity

A CFA was conducted to examine the construct validity of both the PDD and the BMQ Scales. Results of the fit indices for the PDD Scale supported the one-dimension model with all 12 items, except the Chi-square was statistically significant, $\chi_{\left(50\right)}^{2}$ = 124.17, *p* < 0.001, χ^2^/*df* = 2.48, GFI = 0.95, CFI = 0.94, TLI = 0.92, RMSEA = 0.06. The CFA results for the BMQ Scale also supported the one-dimension model with all eight items, except the chi-square was statistically significant, $\chi_{\left(12\right)}^{2}$ =25.20, *p* = 0.01, χ^2^/*df* = 2.10, GFI = 0.98, CFI = 0.98, TLI = 0.96, RMSEA = 0.05.

#### Reliability

Reliability was tested using internal consistency Cronbach's Alpha for the PDD Scale and the modified BMQ-General. The PDD scale consisted of only one factor, and Cronbach's Alpha was 0.85. Cronbach's Alpha of the modified BMQ-General was 0.78.

### Differences in Mental Health and Psychotropic Stigma

Paired samples *t*-test was conducted to examine the difference between the mean scores of the PDD and the BMQ scales. There was a statistically significant difference between stigma toward mental health conditions and stigma toward psychotropic medications with a moderate effect size, *t*_(389)_ = 11.93, *p* < 0.001, Cohen's *d* = 0.60, with significantly higher stigma toward psychotropic medications (*M* = 2.66, *SD* = 0.60) compared to stigma toward mental health conditions (*M* = 3.15, *SD* = 0.60).

### Differences in Stigma by Mental Health Treatment Preferences

A one-way MANOVA was conducted to examine the difference of the PDD and BMQ among participants with different treatment preferences. Descriptive statistics are provided in Table 2. The Box's *M*-test result indicated the equal variance-covariance matrices assumption was met, *p* = 0.25. Using the Wilks' Lambda, the dependent variables were significantly different among students with different treatment preferences with small to moderate effect size, Wilks' ƛ = 0.90, *F*_(6,764)_ = 6.74, *p* < 0.001, η^2^ = 0.05. A follow-up one-way ANOVA was conducted. The Levene's test results indicated the equal variance assumption was met for both PDD and BMQ scales, *p* = 0.94, *p* = 0.06, respectively. Mental health stigma did not significantly differ for students with different treatment preferences, *F*_(3,383)_ = 1.21, *p* = 0.31,. However, stigma toward psychotropic medications did statistically significantly differ for students with different treatment preferences with moderate effect size, *F*_(3,383)_ = 12.29, *p* < 0.001, η^2^ = 0.09. Bonferroni *post-hoc* analysis revealed that students who preferred counseling had significantly less psychotropic stigma than students who preferred both counseling and medication (*p* < 0.001) and those who preferred medication only, *p* = 0.048. Students who do not prefer both treatment methods had significant less psychotropic stigma than students who preferred both methods, *p* = 0.02. Students with other treatment preferences did not significantly differ in psychotropic stigma.

**Table 2:** Participants' stigma scores by treatment preferences.

**Stigma**	**Total** ***N* = 390** ***M (SD)***	**Counseling** ***n* = 163** ***M (SD)***	**Medication** ***n* = 56** ***M (SD)***	**Both** ***n* = 155** ***M (SD)***	**Neither** ***n* = 13** ***M (SD)***
Mental health	3.15 (0.60)	3.16 (0.59)	3.18 (0.61)	3.17 (0.60)	2.85 (0.72)
Psychotropic	2.66 (0.60)	2.84 (0.52)	2.60 (0.64)	2.46 (0.59)	2.95 (0.77)

### Differences in Stigma Between Pharmacy Class

A one-way MANOVA was conducted to examine the difference of the PDD and BMQ among different cohorts. Descriptive statistics was presented in Table 3. The Box's *M*-test result indicated the equal variance-covariance matrices assumption was met, *p* = 0.47. Using the Wilks' Lambda, the dependent variables were not statistically significant different among different cohorts, Wilks' ƛ = 0.97, *F*_(6,770)_ = 1.95, *p* = 0.07.

**Table 3:** Participants' stigma scores by year in Pharm. D. Program.

**Stigma**	**Total** ***N* = 390** ***M (SD)***	**1st year** ***n* = 137** ***M (SD)***	**2nd year** ***n* = 142** ***M (SD)***	**3rd year** ***n* = 98** ***M (SD)***	**4th year** ***n* = 13** ***M (SD)***
Mental health	3.15 (0.60)	3.19 (0.59)	3.03 (0.60)	3.28 (0.61)	3.22 (0.47)
Psychotropic	2.66 (0.60)	2.68 (0.60)	2.66 (0.653)	2.62 (0.54)	2.63 (0.61)

## Discussion

In this cross-sectional survey of student pharmacists, the participant cohort was not associated with differences in stigma associated with mental health conditions and psychotropic medications. Stigma toward psychotropic medications was significantly associated with student preference for mental health treatment, and student pharmacists' psychotropic medication stigma was significantly greater than their mental health stigma.

Health professions students, specifically medical students, have been shown to have stigma toward mental health (28). These results concur with previous research regarding stigma in student pharmacists. While Bell et al. focused on stigma toward severe depression and schizophrenia, the current study looked at stigma toward mental health more broadly (20). The current study indicates that stigma toward psychotropic medications (1) exists and (2) does not lessen with progress through pharmacy school's programs. Practicing healthcare providers have also been shown to have stigma toward mental health conditions (29) further supporting the finding that stigma does not decrease after graduation.

Research on exposure to patients with mental health conditions and the subsequent effect on mental health stigma is varied. Some studies show that repeated exposure does not decrease stigma while others show that it does (20, 30). Some student pharmacists are exposed to patients with mental health conditions over the course of obtaining their degree; however, stigma was still present and unrelated to prior exposure.

A meta-analysis of mental health treatment preference performed in 2013 showed that people generally prefer psychological treatment over pharmacological treatment for mental health conditions (31). This was mirrored in our study, which showed that the majority of student pharmacists preferred psychological treatment for mental health conditions as opposed to other options, including medications, a combination of counseling and medication, or no treatment.

### Implications

This study has several implications. First, given the significant difference between mental health stigma and psychotropic stigma, it seems clear that psychotropic stigma should be studied on its own more extensively to understand how healthcare providers' stigma may affect pharmacist counseling toward patients concerning these medications. Stigma toward mental health conditions is already associated with lower adherence rates (32, 33). However, little research has been done on stigma toward medications specifically, especially psychotropic medications. This study lays the groundwork for future research to focus specifically on stigma toward psychotropic medications and its effects.

This study also demonstrated that current pharmacy programs may be augmented with mental health training to decrease stigma surrounding mental health and psychotropic stigma. The most effective type of intervention to decrease mental health stigma in students is social contact (working with patients who have mental health conditions) (34). Schools of pharmacy allow many opportunities for students to interact with real patients through experiential and co-curricular programs. Being purposeful when selecting those patients so that students have contact with patients who have mental health conditions is one way stigma among student pharmacists may be decreased. With the call for increased pharmacist involvement in screening and treatment of mental health conditions (9, 10), student pharmacists may benefit from educational experiences that affords them experience in this area. Efforts should be made to decrease stigma toward both mental health and psychotropics.

### Limitations

Due to the low number of fourth-year respondents, results related to how stigma is affected over time through pharmacy school should be interpreted with caution. This was likely due to the different formats the survey was delivered in: fourth-year student pharmacists were given an online version of the survey while first- through third-year student pharmacists received a paper survey at the end of class. Low response rate may also have been due to the differences in available time to complete the survey for fourth-year students compared to first-, second-, and third-year students, given that schedules are less flexible in the fourth year.

Social desirability bias is a concern for this study since stigma is a sensitive topic especially among healthcare providers who are expected to give fair and unbiased treatment to all patients. Care was taken to make sure responses were anonymous with each student being given an envelope to put their survey into after completing it, so students could feel comfortable choosing not to participate in the study or feel secure that if they did fill out the survey, their responses would not be linked back to them.

## Future Research

Future research should focus on how these mental health and psychotropic stigmas affect student pharmacists' care of patients who have mental health conditions and take psychotropic medications. Several questions depended on the perceptions of each student. For example, “psychotropic medications” included a wide range of medications. Some psychotropic medications have more adverse reactions and side effects than others, and people may have more negative connotations toward these differing psychotropic medications. Performing a qualitative study to delve into student pharmacists' perceptions of psychotropics and mental health would be useful in understanding their responses to the present survey.

## Conclusion

Student pharmacists demonstrated greater psychotropic stigma compared to mental health stigma, and their mental health treatment preference is associated with stigma toward psychotropic medications. Understanding how these stigmas affect their decisions toward patients should be researched further.

## Data Availability Statement

The raw data supporting the conclusions of this article will be made available by the authors, without undue reservation.

## Ethics Statement

The studies involving human participants were reviewed and approved by the Institutional Review Board at Auburn University. Written informed consent was obtained for participants who completed paper surveys. It was not required for those completing online surveys; instead, an information letter was provided at the beginning of the survey.

## Author Contributions

Design, data collection, and drafting were done by BD, CM, and KG. Data analysis, critical revision, and final approval of article were completed by BD, CM, C-hW, and KG. All authors contributed to the article and approved the submitted version.

## Funding

BD was funded by a CCTS Predoctoral Clinical/Translational Research Program (TL1) (grant # 1TL1TR003106-01) and the American Foundation for Pharmaceutical Education (AFPE). CM was supported by the American Foundation for Pharmaceutical Education (AFPE) and the PhRMA Foundation.

## Conflict of Interest

The authors declare that the research was conducted in the absence of any commercial or financial relationships that could be construed as a potential conflict of interest.

## Publisher's Note

All claims expressed in this article are solely those of the authors and do not necessarily represent those of their affiliated organizations, or those of the publisher, the editors and the reviewers. Any product that may be evaluated in this article, or claim that may be made by its manufacturer, is not guaranteed or endorsed by the publisher.
